# Myosin cluster dynamics determines epithelial wound ring constriction

**DOI:** 10.1016/j.isci.2025.113030

**Published:** 2025-06-30

**Authors:** Alka Bhat, Rémi Berthoz, Simon Lo Vecchio, Coralie Spiegelhalter, Shigenobu Yonemura, Olivier Pertz, Daniel Riveline

**Affiliations:** 1 Laboratory of Cell Physics ISIS/IGBMC, CNRS and University of Strasbourg, Strasbourg, France; 2 Institut de Génétique et de Biologie Moléculaire et Cellulaire, Illkirch, France; 3 Centre National de la Recherche Scientifique, UMR7104, Illkirch, France; 4 Institut National de la Santé et de la Recherche Médicale, U964, Illkirch, France; 5 Université de Strasbourg, Illkirch, France; 6 Department of Cell Biology, Tokushima University Graduate School of Medicine, Tokushima, Tokushima 770-8503, Japan; 7 Institute of Cell Biology, University of Bern, Baltzerstrasse 4, 3012 Bern, Switzerland

**Keywords:** Cell biology, Organizational aspects of cell biology

## Abstract

Collection of myosin motors and actin filaments can self-assemble into submicrometric clusters under the regulation of RhoA. Emergent dynamics of these clusters have been reported in a variety of morphogenetic systems, ranging from *Drosophila* to actomyosin assays *in vitro*. In single-cell cytokinetic rings, actomyosin clusters contribute to stress generation when their dynamics are radial, and they facilitate transport when their dynamics are tangential to the direction of ring closure. Here, we show that these phenomena hold true for actomyosin multi-cellular rings during wound closure in epithelial monolayers. We assessed the activity of RhoA using FRET sensors, and we report that cluster dynamics does not correlate with RhoA activity. Nevertheless, we show that bursts of RhoA activation precede recruitment of myosin. Altogether myosin clusters dynamics is conserved between single and multi-cellular systems, and this suggests that they could be used as generic read-outs for mapping and predicting stress generation and shape changes in morphogenesis.

## Highlights

• Myosin clusters appear in multicellular actomyosin ring
• Two types of moving clusters co-exist: tangential and radial clusters
• Their proportions are determinant for the speed of ring closure
• Clusters dynamics could serve as readout for stress distribution in tissues

## Graphical abstract

**Figure:**
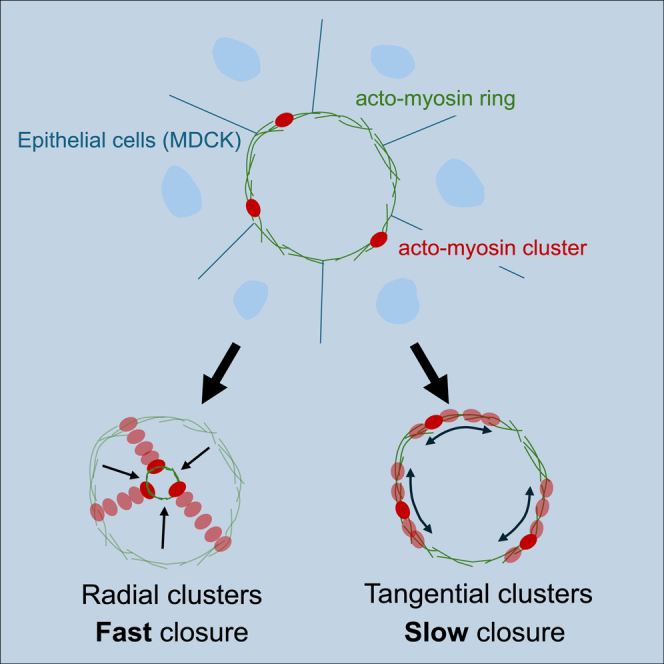

## Introduction

Morphogenesis follows a highly dynamic orchestration of cellular phenomena.^1^^,^^2^ Cells modify their shapes, divide, delaminate, and change their localizations with their neighbors. These transformations in multicellular systems have been the focus of recent studies at the interfaces between Physics and Biology. Generic rules for self-organization are expected to emerge by studying and comparing similar shape transformations in different biological systems.

The actomyosin cytoskeleton is a key player in shape transformations within multicellular systems.^3^^,^^4^^,^^5^ Actomyosin is composed of arrays of myosin II motors interacting with actin filaments.^6^ This network of actomyosin, which contracts and expands, is based on the position and orientation of actin filaments and myosin motors.^7^ Actomyosin networks can organize in different architectures, playing different roles in cell and tissue dynamics.^3^ Also the small RhoA GTPase regulates activity of myosin through signaling pathways well documented as well as with their GEF/GAP regulatory switches.^8^^,^^9^ Their dynamics and their connections to myosin activities are important and they can mediate changes in shapes of the cytoskeleton and cell shapes.^6^

To predict mesoscopic outcomes from the cytoskeleton organization, molecular composition information is not sufficient, because thousands of individual molecules interacting together result in collective behavior^10^ which do not result from the simple addition of individual behaviors. Also, actomyosin network architectures are often not known (and distinct from sarcomeric organizations) and they can be diverse for different *in vivo* systems.^6^

In mammalian and fission yeast cells, the dynamics of long-lived *myosin clusters* measuring roughly 200 nm in size was shown to be a readout fully capturing the contraction of the actomyosin ring.^10^ Ring constriction showed two different types of cluster dynamics where myosin clusters were observed to be *rotating* clockwise and counter-clockwise independently in the case of fission yeast rings at a velocity of 2 μm/min, whereas they remained *still* within the ring framework in the case of mammalian cells. Theory and experiments using specific inhibitors associated *rotating clusters* with transport of the wall machinery for fission yeast, and *still clusters* to stress generation and constriction of the ring in mammalian cells. These rules should also be relevant for other actomyosin systems found in other situations. In particular, rings in multicellular system as well are expected to show these clusters dynamics with a similar outcome, according to the genericity of associated theoretical framework for active fluids.^11^

In this context, we evaluate the dynamics of self-organized myosin clusters in an epithelial monolayer. To test whether the dynamics of myosin clusters in multicellular systems is similar to their dynamics in single/isolated cells, we generated controlled actomyosin wound rings in epithelial monolayers,^12^^,^^13^ a geometry that allows comparison with the cytokinetic ring in single cells. Two classes of cluster dynamics, still and rotating, are associated with stress generation and transport respectively. We compared both dynamics throughout different configurations and we made correlations with the associated RhoA activity. Although RhoA activity correlates with cluster self-assembly, there is no relationship between cluster behaviors and RhoA levels. Finally, we propose that spatiotemporal dynamics of actomyosin clusters could be used as a readout for stress generation in tissues.

## Results

### Velocity of wound closure is independent of cell number around the wound

**Figure 1:**
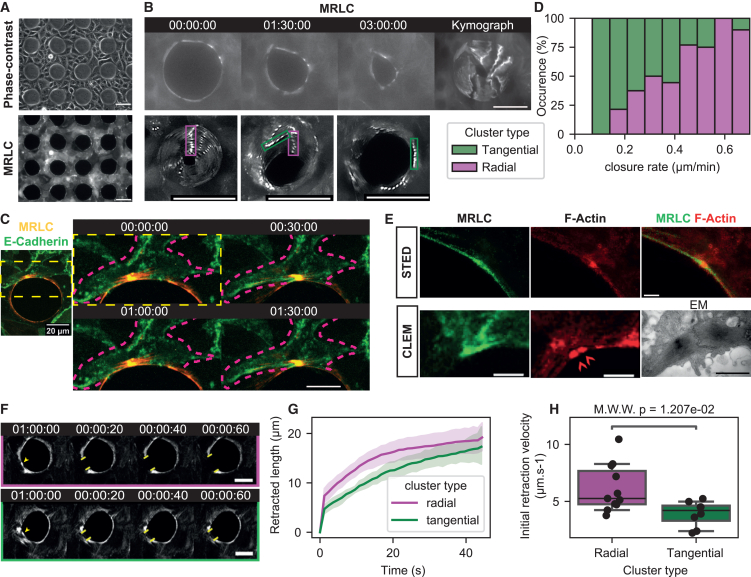
Myosin clusters in the actomyosin cable drive wound closure, radial clusters generate stress (A) Wounds are created in an epithelial sheet by attaching micro-fabricated PDMS pillars on the microscope slide. After cell seeding, an epithelial sheet forms between pillars. We can image the setup with fluorescence microscopy and phase-contrast. Scalebars represent 50 μm. (B) The PDMS pillars can be detached from the microscope slide, and the wound closes. We observe myosin clusters during wound closure (snapshot series, left), and generate kymographs to track their motion (right and bottom). We classify them in two categories: radial clusters (green boxes) do not move along the periphery of the wound, contrary to tangential clusters (violet boxes). Scalebars represent 50 μm. (C) Myosin clusters cannot cross cell-cell junctions at the wound ring. We illustrate a representative example of a dynamic cluster trapped between two cell-cell junctions (marked by E-Cadherin). Overview of the wound ring (left) and time lapse of cluster dynamics (right). The cluster is trapped between the cell-cell junctions. Scalebar represents 20 μm. (D) The percentage of radial clusters on a wound ring correlates with the velocity of wound closure, suggesting that radial clusters contribute more to wound closure (data from 25 wounds, 76 clusters). (E) Super resolution images of myosin clusters. Stimulated emission depletion (STED, top) of phosphorylated myosin and actin near a cluster. Correlative light and electron microscopy (CLEM, bottom) of two clusters around a cell-cell junction with the MRLC-GFP cell line. Red arrows on the F-Actin image indicate the position of the two clusters that are enlarged on the EM image. Scalebar in fluorescence image represents 2 μm, scalebar in EM image represents 1 μm. (F) Time lapse of laser ablation experiments on a radial (top) and tangential (bottom) clusters. Upon ablation (yellow arrow at time 1:00:00 indicates ablation point), the actomyosin cable snaps open (yellow bars delimitate actomyosin cable opening). Scalebar represents 20 μm. (G) We quantify the retracted length as a function of time, post-ablation. Cable portions with radial clusters retract faster than portions with tangential clusters (solid bar and shaded area represent mean ±95% CI from 11 ablations on radial clusters, 8 ablations on tangential clusters). (H) Box-plots of initial retraction velocity measured for radial and tangential clusters, between the timepoints (boxplot dashed solid represents mean value, box height represents quartiles and whiskers represent 95% CI, black dots represent individual data points, from 11 ablations on radial clusters, 8 ablations on tangential clusters). All timecodes are indicated in hh:mm:ss.

To generate wounds, we attached micro-pillars on a substrate and seeded cells in-between. Removal of the micro-pillars generated holes in an epithelial monolayer which eventually closed through the assembly of an actomyosin ring at the frontier region (Figures 1A, S1A, and S2A, see Material and Methods for details). The ring diameter was fixed to 50 μm to rule out closure driven by lamellipodia growth.^13^ We then tracked wound closure and myosin cluster dynamics over time. The velocity of wound closure was constant over the first 100 min (Figure S2B), and the wound then completed closure over 400 min.

To address the contribution of cell number during wound closure, two different approaches were taken: (1) variation of inter-distance between pillars, introducing different numbers of cells between rings (Figure S2A (i-iii), Video S1) and (2) wound closure of a doughnut shape ring to rule out the contribution of cells away from the ring to the closure (Figure S2A’(i-ii) and S1B (i-v)). We measured the different dynamics corresponding to each situation (Figures S2B and S2C). Velocities and dynamics were similar across configurations, suggesting that cells between rings do not play a key role in closure. This suggests that the ring, and not the surrounding tissue, is the main stress generator during closure.

Video S1. Closure of micro-fabricated MDCK rings with a diameter of 50μm diameter (D50) Interdistance between rings is controlled, with 30μm, 100μm and 200μm respectively. Phase contrast and fluorescence (MRLC-GFP) images are shown. Time in hh:mm:ss.

### Myosin density in the ring is constant over time during wound closure

Next, we looked at actomyosin around the wound edge. Specifically, we looked at the *actomyosin cable* which surrounds the epithelial edge as a continuous structure containing 1.8–2 times higher myosin levels than the epithelium base level (Figure S3A (i)). We measured myosin levels in the cable with time: the total myosin intensity was shown to decrease during constriction, keeping the concentration (mean intensity) constant (Figure S3A (ii)). To test whether the abundance of myosin in the whole cell or only along the cable decrease, we also measured the total amount of myosin in cells contributing to the cable (Figure S3B). We find that the total amount of myosin in a cell is constant on the timescale of wound healing, but re-localized to maintain a constant concentration in the cable with a high turnover evaluated by fluorescence after photobleaching experiment (Figure S3C, Materials and Methods). We measured the distance from the actomyosin cable to the substrate by correlative light electron microscopy (CLEM) to be within 0.9–1 μm. In the same experiment, we measured the thickness of the actomyosin cable to be 0.5 μm (Figure S4, Video S8 and S9). Altogether, the ring constricts with constant actomyosin density in the cable leading to wound closure.

### Myosin self-organizes in clusters within the actomyosin cable and exhibits two types of dynamics

We observe *myosin clusters* of about 1 μm in size along every wound ring perimeter spaced by about 10 μm (Figures 1B and 1E, see Material and Methods). In general, we observe 3 to 4 clusters per ring for typically 6 cells with constant myosin concentration and density on the cable (Figure S5A and S5B). Clusters can move along the cable but do not cross cell-cell junctions (Figure 1C). Their motion extends typically micrometer which supports the idea of an active motion.^10^ Also, clusters can collide and fuse (Video S1), as we also reported in our former study on the cytokinetic ring closure.^10^To gain higher resolution images we imaged these clusters with stimulated emission depletion (STED) microscopy and CLEM and measured 500 nm for clusters dimension (Figure 1E), a size which is consistent with previous work in the context of cytokinesis.^10^

We observed cluster dynamics and categorized them in two types (Figures 1B and 1D). Clusters with a constant angular position as the cable constricts are named *radial clusters*. Conversely, clusters with angular motion along the cable perimeter are named *tangential clusters* (Figure 1B and Video S2). Cables could present simultaneously radial and tangential clusters. We observed rare occurrences of myosin clusters transitioning from one dynamic to the other, but most did not. The typical velocity of radial and tangential clusters was evaluated to be 0.05 μm/min and 0.2 μm/min respectively (Figure S5C (ii)). Interestingly, the non phosphorylatable AA strain had less clusters (see Figure S6), consistent with former report from Watanabe et al*.*^14^ Together with immunofluorescence staining of clusters with phospho-myosin antibodies (see Figure 1E STED), this suggests that phosphorylation of myosin is required.

Video S2. Radial and tangential cluster dynamics during constriction Fluorescence images of MRLC-GFP are shown. Time in hh:mm:ss.

### Radial clusters drive fast wound closure

To test whether the dynamics of clusters could be correlated to wound closure, we plotted the constriction velocity as a function of tangential and radial cluster fraction (Figure 1D). We saw a strong correlation: faster constrictions correlated with higher fraction of radial clusters (Figures 1D and S7 Pearson’s R = 0.80). Importantly there was no particular synchronization or oscillation between clusters. Also, we observed that clusters radial motion was associated to a reduction in their inter-distance and closure of the ring (Figure 1B). This suggests that stress could be generated within the cable. Along this hypothesis, it is interesting to note that cell-cell junctions are deformed along local accumulation of clusters as seen by EM images (see Figure S8). We thus propose that radial clusters are associated with stress generation.

**Figure 2:**
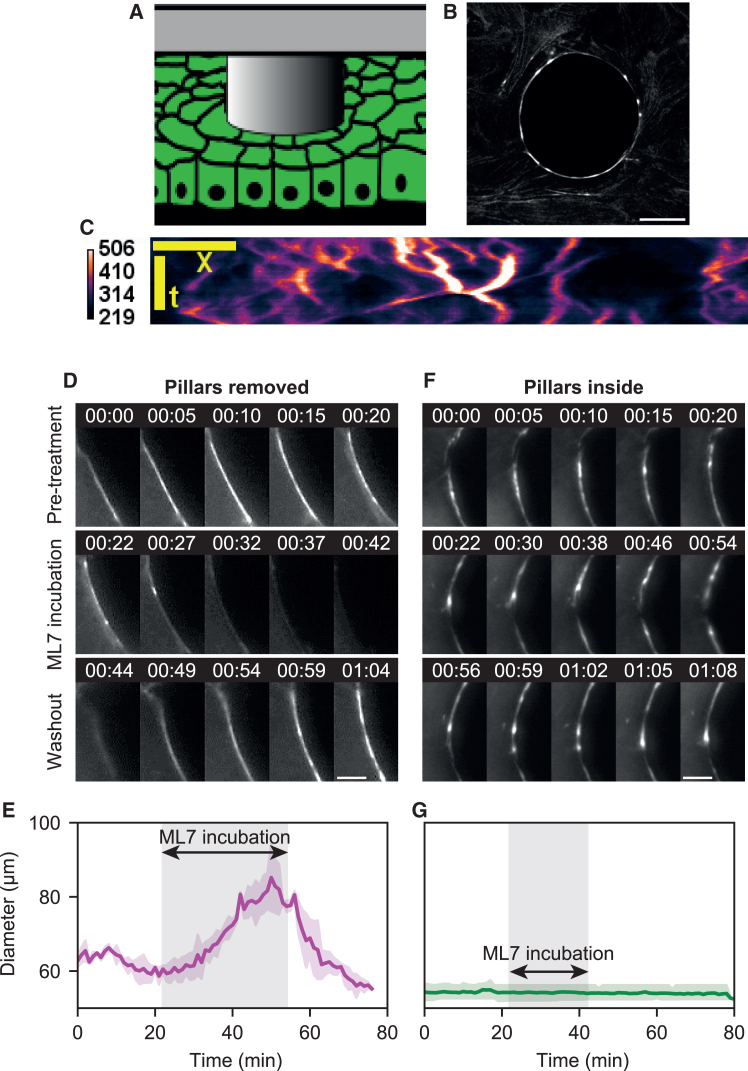
Myosin activity in clusters is responsible for stress generation (A) Scheme and (B) myosin fluorescence image of experiments with the PDMS pillars left inside the monolayer. In this configuration, the wound cannot close. Scalebar represents 10 μm. (C) Kymograph of cluster dynamics around the wound with the pillar left inside. Myosin fluorescence intensity is color-coded. All clusters are tangential. (D) Fluorescence images of a perturbation experiment with myosin inhibitor ML-7 and the pillar removed from the monolayer. Pre-treatment, clusters are radial. During treatment all clusters disappear, as well as the actomyosin cable itself. Post-treatment, the cable and clusters reform. Scalebar represents 5 μm. (E) Graph of the wound diameter during experiments like shown in (D). During treatment, the wound opens. Upon washout (post-treatment), the wound closes to reach the diameter it had prior treatment (solid line represents mean, shaded areas represent 95% CI from 3 wound rings; vertical y axis scale is shared with panel (D)). (F) Fluorescence images of a perturbation experiment with myosin inhibitor ML-7 and the pillar left inside the monolayer. Pre-treatment, all clusters are tangential. During treatment, clusters continue to move, the wound does not change diameter. Post-treatment, the same dynamics continue. Scalebar represents 5 μm. (G) Graph of the wound diameter during experiments like shown in (F). During treatment, the wound does not change diameter (solid line represents mean, shaded areas represent 95% CI from 3 wound rings; vertical y axis scale is shared with panel (E)).

To test whether we could control the dynamics of clusters externally, we left PDMS micro-pillars inside the cell monolayer as a physical barrier to prevent wound closure (illustration Figures 2A and 2B, Video S6). As expected, we did not observe closure. Surprisingly, all clusters moved around the ring with a tangential dynamic (kymograph Figures 2C and 2F). This consistent with the trend of Figure 1D as all clusters are tangential when the closure is stalled.

To rule out the potential contribution of cell motion to cluster dynamics, we measured positions of myosin clusters with respect to the associated cell-cell junction (Figure S9A). This analysis revealed that movement of myosin clusters occurs within the cable independently of the rest of the cell (Figures S9B and S9C). Altogether, cluster dynamics correlate with wound closure dynamics.

### Radial clusters generate more stress than tangential clusters in the actomyosin cable

We used laser ablation to evaluate the stress associated with radial and tangential clusters. Upon ablation, wound constriction stalls and the ring immediately opens, showing that the cable is under tension (Figures 1F and 1G). Ablation of radial clusters leads on average to an initial retraction velocity 1.6 times larger than of the ones measured for tangential clusters (Figure 1H, Videos S4 and S5). Retraction velocities corresponding to radial and tangential clusters were evaluated to be 6.2 ± 0.6 μm/s and 3.9 ± 0.4 μm/s respectively (Mean ± SEM, Figure 1H, 19 clusters, Mann-Whitney U test’s *p*-value = 0.012), much faster than retraction at cell-cell junction.^15^ Assuming that viscous friction within the actomyosin gel is the same throughout the cable, we interpret changes in retraction velocity as changes in tension,^16^ consistent with our former characterization of clusters.^10^^,^^17^ These results indicate that radial clusters have higher tension associated with them compared to tangential clusters.

Video S4. Ablation of a tangential cluster Cells express MRLC-KO1 (red) and E-cadherin mNG (green). Time in hh:mm:s.

Video S5. Ablation of a radial cluster Cells express MRLC-KO1 (red) and E-cadherin mNG (green). Time in hh:mm:ss.

### Myosin activity in clusters is responsible for stress generation

To test the contribution of myosin clusters to stress generation, we used the potent reversible myosin light-chain kinase inhibitor drug ML-7.^14^ Exposing the actomyosin rings to the kinase inhibitor led to the disappearance of myosin clusters and the actomyosin ring (Figure 2D) – as in the presence of the Rho kinase inhibitor ROCK (Figure S10). This disappearance was simultaneous with an increase in ring diameter (Figure 2E). After washout of the kinase inhibitor, actomyosin structures reappeared, and the cable restarted constriction (Figure 2D). This demonstrates myosin activity is necessary and sufficient for ring constriction and the formation of myosin clusters (radial as well as tangential). In addition, disappearance and reappearance of clusters upon inhibition and reactivation of the kinase support additional evidence regarding the self-organized nature of myosin clusters. Similarly, the kinase inhibitor was used in a configuration with pillars left inside the monolayer (Figure 2F). In this configuration, wounds do not close, and clusters are all tangential. Surprisingly, during drug exposure these clusters did not disappear, and the tangential dynamic was unaffected in contrast to control condition (compare Figures 2D and 2F), suggesting a mechanosensing response of myosin clusters in the presence of a physical barrier.

### Cluster types are independent of RhoA activity

**Figure 3:**
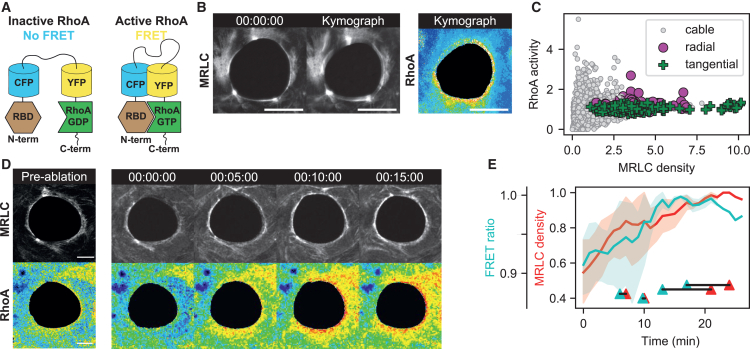
RhoA activity near clusters is not associated with cluster dynamics (A) To measure RhoA activity, we used a RhoA FRET biosensor. See Material & Methods for details. (B) Fluorescence image of myosin clusters on a wound ring (left), kymograph showing their dynamics and wound closure (center), and the kymograph for the RhoA activity of the same sequence (right). Scalebar represents 25 μm. (C) Time-lapse of ablation experiments with fluorescence readouts for myosin and RhoA activity. On this timescale, the ablated cable reforms a complete actomyosin cable associated with a burst in RhoA activity (ablation is performed on the cluster indicated by the yellow arrow in the first panel). Scalebars represent 10 μm. (D) Graph of RhoA activity as a function of myosin density measured in short sections of the actomyosin cable on clusters of both types (green crosses, violet dots) and on the cable (gray dots). When myosin density is high, RhoA activity plateaus to an average value. This plateau is where all crosses corresponding to clusters are located in the graph. Radial (violet) and tangential (green) clusters are located together in this region (points are collected from 13 radial clusters, 36 tangential clusters, on 16 wound rings). (E) RhoA activity (green) and myosin density (red) temporal evolution after ablation, at the timescale of cable reformation. RhoA activity peaks before myosin density (solid lines and shaded areas indicate Mean ± SEM from 5 ablations). Joined triangles at the bottom of the graph indicate the peak RhoA activity and myosin density for individual experiments, showing the temporal advance of RhoA density peaks on myosin density peaks.

Local transient increase of RhoA activity has been reported to occur in response to wounds in single cells as well as multicellular systems.^18^^,^^19^^,^^20^To assess involvement of the RhoA pathway and its spatiotemporal activity throughout the actomyosin wound ring, we mapped RhoA activity with sensors (Figures 3A and S11),^21^ characterized in a variety of situations.^14^^,^^21^ The highest RhoA activity is found at the wound boundary where the actomyosin cable is assembled (Figures 3B and 3C). Upon laser ablation, an increase in RhoA activity precedes myosin recruitment by 4 ± 2 min (Mean ± SEM, Figures 3C and 3E, Video S7).

Video S6. Cluster dynamics in non-closing rings with pillars left inside the monolayer Phase contrast and fluorescence (MRLC-GFP) images are shown. Time in hh:mm:ss.

Video S7. Ratiometric FRET analysis during myosin recruitment after ablation Both MRLC-mCherry and RhoA FRET channels are shown. 1 min interval between images for a duration of 37min. Time in hh:mm:ss.

Video S8. EM serial sections with 80 nm thickness of a ring portion, illustrating the thickness of the ‘ring with cluster’ to be ∼0.5 μm

Video S9. Other examples of clusters by EM

To test the correlation between RhoA activity and cluster dynamics, we measured myosin density and RhoA activity along the rings. We grouped RhoA activity measurements to the type of clusters (Figure 3D). We found that increased RhoA activity in rings correlates with increased myosin concentration for low values of myosin concentration, and thus we observe higher RhoA activity in clusters than in the actomyosin cable. Nevertheless, all clusters - radial and tangential - share similar levels of high RhoA activity (Figure 3D). This suggests that cluster dynamics do not depend on RhoA levels but possibly emerge through self-organization of the myosin lattice in response to different forces downstream of RhoA-ROCK-MLC signaling.

## Discussion

In this study, we report that actomyosin clusters self-organize in multicellular systems and exhibit properties similar to clusters in cytokinetic rings. Radial clusters correlate with faster closure and generate higher stress in contrast to tangential clusters, as shown by our laser ablation experiments. RhoA activity is high within the actomyosin cable and constant in clusters. This suggests that clusters can self-organize as an active gel with no regulation, as suggested by previous theoretical work.^22^

It is interesting to note that myosin clusters are reported in a variety of model systems *in vivo*. They exhibit similar dynamics as the clusters we report. In *Drosophila,* myosin clusters undergo oscillations similar to our radial clusters and this dynamics is associated with deformation of amnioserosa cells.^23^ In *Zebrafish* and mice, continuous motion of myosin clusters is related to tissue flow.^24^^,^^25^ This also resembles self-organized clusters also reported in *in vitro* assays with purified actin associated proteins.^26^

Cluster dynamics could serve as a readout for force generation in the developing embryo.^23^ The mesoscopic nature of clusters with thousands of molecular actors prevents the unique molecular description but supports the approach where clusters are treated as generic entities like particles. Combining cluster readout and their tracking over time with cell shape would bring a local fiducial marker to map fluctuations in shapes and in tissue dynamics. An analysis pipeline could map in space and time the clusters dynamics. Their trajectories would be converted into stress generation with potential predictive power for morphogenetic events^27^ to be tested in the future. A combination of arguments substantiates the fact that radial clusters are the key stress generators: inhibition of myosin activity leads to arrest of closure together with disappearance of clusters consistent with our former study on cytokinetic ring^10^; the speed of closure is proportional to radial clusters within the ring; cell-cell junctions are locally deformed. It is worth noting that we could see in the EM data that local cell-cell deformation in the vicinity of the clusters at junctions (Figure S8), which further supports the notion that local tensional stress could operate. Future work at larger resolution will be needed to evaluate each stress component.

In this context it is worth noting that such active gels in the vicinity of a membrane can produce chaotic dynamics.^22^ This resembles closely the continuous back and forth movement of clusters when the ring is constrained by a fixed pillar. We propose that radial clusters are associated to stress generation perpendicular to the interface when pillars are removed. It would be interesting to measure tangential and radial stresses by using deformable pillars with controlled elasticity to support this framework.

The persistence of clusters during ML7 incubation with pillars inside the tissue was not expected since myosin clusters disappeared in the absence of pillars. This calls for a potential mechanosensory mechanism. Along the idea of radial and tangential stress generation, we propose that myosin clusters submitted to the resisting force of the pillar could compensate for the expected effect of the inhibitor and still undergo motion. It would be interesting to test this mechanism by quantifying the phosphorylation of myosin in live experiments to determine the relevance of our hypothesis. This could also allow to determine whether other kinases could be involved in the myosin II activation/dynamics such as Ca^2+^-dependent myosin light-chain kinase or myotonic dystrophy kinase-related Cdc42-binding kinases.

Our results also show that RhoA can mechanosense the wound to get activated and increase ROCK-MLC signaling. Self-organization of myosin yields the clusters dynamics in contrast to RhoA spatially regulating the myosin arrays. This is understandable since RhoA is a lipidated molecule (geranyl geranylated) and thus diffuse in the plasma membrane. This explains why a RhoA activity appears to be higher on average around the cable. However, myosin clusters whether radial or tangential exhibit the same RhoA activity. As a result, we propose that both dynamics are controlled by generic properties of actomyosin interacting systems, well captured by the formalism of pure active gels without regulation.

Altogether, the physics of force generation by myosin clusters appears to be conserved in cytokinesis and in wound closure in multicellular system. But this dynamic appears to be conserved also in larger scales. It was reported^28^ that multicellular rings exhibit dynamic of cell clustering which is very reminiscent of our observations, three orders of magnitude higher in dimension than our myosin clusters. Specifically, groups of cells within a ring appeared to exhibit collective contraction and flows. This suggests that the specificities of each contractile systems are not essential. Continuous contractile actomyosin systems leading to local force dipoles share generic behaviors, from the cytokinetic ring of single cells to a collection of epithelial contractile cells. This invariance in scale is consistent with the genericity of active matter theory.^29^ It will be interesting to further test myosin clusters dynamics across scales to characterize morphogenetic events.

### Limitations of the study

Future studies will be needed to distinguish between passive and active stresses as well as between isotropic and anisotropic stresses. Also, it will be interesting to evaluate how myosin activity contributes to uniaxial stress along the cable. In our study, this cable is treated as a continuum and additional studies will allow to evaluate transition between series of cellular segments and an actual cable as reported in our cytokinetic ring work.^10^ Finally, it will be interesting to characterize the potential roles of substrate (flexibility and coatings) in the distribution of force transmission together with 3D imaging.

## Resource availability

### Lead contact

Further information and requests for resources and reagents should be directed to and will be fulfilled by the Lead Contact Daniel Riveline, riveline@unistra.fr.

### Materials availability

This study did not generate new unique reagents.

### Data and code availability

• Data: The data are available upon request.
• Code: Original/custom code for FRET analysis was developed for this study and is available from the authors upon request.
• Any additional information required to reanalyze the data reported in this paper is available from the lead contact upon request.

## Acknowledgments

We thank Karsten Kruse (University of Geneva) for insightful discussions and the Riveline team for feedback and suggestions. We thank E. Grandgirard and E. Guiot and the Imaging Platform of IGBMC. We are grateful for experimental support from Alf Honigmann (MPI-CBG Dresden) and Kobus van Unen (University of Bern). A.B. is supported by the 10.13039/501100003768University of Strasbourg and R.B. is supported by IMCBio. D.R. acknowledges the Interdisciplinary Thematic Institute IMCBio, part of the ITI 2021–2028 program of the University of Strasbourg, CNRS and Inserm, which was supported by IdEx Unistra (ANR-10-IDEX-0002), and by SFRI-STRAT’US project (ANR 20-SFRI-0012) and EUR IMCBio (ANR-17-EURE-0023) under the framework of the French Investments for the Future Program. D.R., R.B., and O.P. are supported by 10.13039/501100001711SNSF Sinergia grant CRSII5_183550.

## Author contributions

D.R. designed and supervised the study. A.B. performed the experiments with support from R.B. and S.L.V. C.S. performed the EM, O.P. contributed to the FRET, S.Y. prepared some cell lines. All authors contributed to the writing of the MS.

## Declaration of interests

The authors declare no competing interest.

## STAR★Methods

### Key resources table

**Table:** 

REAGENT or RESOURCE	SOURCE	IDENTIFIER
**Antibodies**		

Goat anti mouse IgG, Star Red	Abberior	Cat# 2-0002-011-2; RRID: AB_2810982
Goat anti rabbit IgG, Star Red	Abberior	Cat# STRED-1002-500UG, RRID: AB_2833015

**Biological samples**		

Fetal Bovine Serum	HyClone	10309433

**Chemicals/drugs, peptides, and recombinant proteins**		

ML-7	Sigma-Aldrich	M-1296
Chlorotrimethylsilane (TMCS)	Sigma-Aldrich	92360
Y27632 (ROCK inhibitor)	Sigma-Aldrich	Y0503
Lipofectamine^TM^ 2000	Invitrogen	11668500
Penicillin-Streptomycin	Gibco	11548876
Pluronic™ F-127	Invitrogen	P6866
PDMS (Polydimethylsiloxane))	Sylgard	DC-184
DMEM (1g/L glucose)	Gibco	31885049
Leibovitz L-15 media	Gibco	11540556
Phalloidin Star Red	Abberior	STRED-0100
TRITC-fibronectin	Cytoskeleton inc.	FNR02

**Experimental models: Cell lines**		

MDCK WT (Wild type)	Prof. S Yonemura, RIKEN, Japan	Watanabe et al.^14^
MDCK-MRLC GFP (Wild type myosin light chain-GFP)	Prof. S Yonemura, RIKEN, Japan	Watanabe et al.^14^
MDCK MRLC KO1 Ecad mNG	Prof. S Yonemura, RIKEN, Japan	Watanabe et al.^14^
MDCK FRET Donor: acceptor (CFP/YFP)	Olivier Pertz Lab, UniBe, Switzerland	Pertz et al.^30^
MDCK FRET Donor acceptor (CFP/YFP) MRLC mCherry	Riveline Lab, IGBMC, France	–

**Software and algorithms**		

ImageJ (FIJI)		
MATLAB		
PIVLab		
Micromanager software		

### Experimental model and study participant details

#### Cell lines and culture conditions

We used the following Madin-Darby Canine Kidney (MDCK II) cell lines in this study: MDCK RhoA FRET, fused to MRLC:mCherry. We established a stable MDCK cell line expressing the RhoA FRET biosensor^30^ using PiggyBac transposon-mediated engineering. Cells were co-transfected with the biosensor plasmid and a transposase. Following genomic integration, fluorescent cells were isolated by flow cytometry and subsequently maintained under antibiotic (Hygromycin B) selection for three weeks. Subsequently the selected cell repertoire was transfected with MRLC:mCherry. MDCK wild-type cells expressing wild type Myosin Regulatory Light Chain (MRLC) fused to GFP (MRLC::GFP) and cells co-expressing MRLC fused to Kosabira Orange (MRLC::KO1) and E-cadherin fused to Neon-Green (Ecad:mNG). We also characterized clusters with the non phosphorylatable MRLC AA-GFP.^14^ The cell lines were confirmed to be free of mycoplasma contamination. Cells were cultured at 37°C with 5% CO_2_ in DMEM (1 g/L) with 10% Fetal Bovine Serum (FBS HyClone 10309433) and 1% penicillin-streptomycin (Gibco 11548876)*.* For imaging, we used L-15 medium (Invitrogen) supplemented with 10% FBS and 1% penicillin-streptomycin. For experiments with myosin inhibitors, we used ML-7 (#I2764, Sigma) at a concentration of 40 μM. For immunofluorescence microscopy, we used our standard protocol and antibodies.^10^ We used the following inhibitors, ROCK (50 μM, #Y0503, Sigma) and ML7 (100μM, #M-1296, Sigma).

### Method details

#### Microfabrication and cell plating

The protocol was adapted from Vedula et al.^13^ Briefly, SU-8 pillars with a diameter of 50 μm and height of 80 μm were micro-fabricated on silicon wafers using UV photolithography. Polydimethylsiloxane (PDMS:cross-linker with a ratio of 9:1) was poured on the silicon wafer with 3D patterns and left at 65°C overnight for curing. The PDMS block with pillars was bonded to the glass coverslip using oxygen plasma, and the assembly was left at room temperature for 40 to 60 min. Next, to avoid attachment of cells to the PDMS pillars, the sample was incubated with Pluronic acid (0.2% in double distilled water) for 40 to 60 min for passivation. Then the liquid was removed, and the sample was left to dry. Next, cells were plated at a concentration of 21000 cells/μL inside the sample by inoculating 20 μL of cell suspension close to the entry point below the PDMS. Cells entered by capillarity and the available volume was completed with serum containing media. The sample was incubated at 37°C with 5% CO_2_ for 14 to 15 h. Unless otherwise mentioned in the main text, after cells spread evenly around the motifs, the PDMS block was carefully removed leaving controlled wounds in the monolayer.

#### Correlative light and electron microscopy (CLEM)

MDCK cells were cultured on laser micro-patterned Aclar support.^31^^,^^32^ Fluorescent microscopy images were first acquired on an inverted Leica confocal microscope (SP5) equipped with a Z-galvo stage with temperature control (whole microscope). A standard or resonant scanner (8000 Hz, 8000 lines/s) was used for fast imaging. DPSS 561 (561 nm, 10%) and argon (488 nm, 10%) laser were used for excitation of cells expressing, MRLC-KO1 and Ecad-mNG respectively. CX PL APO 40x/1.25–0.75 OIL objective was used for acquisition. The acquisition was done with photomultiplier tube (PMT) detectors at an interval of 1 min. Acquisition was followed immediately by fixation of cells and fragments using 2.5% glutaraldehyde and 2.5% formaldehyde in 0.1M cacodylate buffer for 2 h at 4°C. After washing, the samples were post-fixed for 1 h in 1% osmium tetroxide [OsO4] reduced by 0.8% potassium hexacyanoferrate (III) [K3Fe(CN)6] in H2O at +4°C. After extensive rinses in distilled water, samples were then post-stained by 1% uranyl acetate for over-night at +4°C and rinsed in water. Samples were dehydrated with increasing concentrations of ethanol and embedded with a graded series of epon 812 resin. Samples were finally polymerized at 60°C for 48 h. Ultrathin serials sections (80nm) were picked up on 1% pioloform coated copper slot grids and examined using a Philips CM12 TEM electron microscope (Philips, FEI Electron Optics, Eindhoven, Netherlands) operated at 80kV and equipped with an Orius 1000 CDD camera (Gatan, Pleasanton, USA).

#### Optical setups for imaging of wound rings

For acquiring temporal dynamics in multicellular rings for phase contrast and fluorescence imaging, epifluorescence inverted Olympus CKX41 setups were used. All the microscope setups were enclosed inside a temperature-controlled chamber stably maintained at 37°C. The samples were mounted either on a Marzhauser stage with a stepper motor or with Tango controller, enabling multi-position acquisitions. The optical path to the samples was controlled with the help of shutters by (Uniblitz VCM-D1/VMM-D1 or ThorLabs SC10) to prevent phototoxicity in case of continuous exposure. A fluorescence excitation lamp (Leica E6000) with mercury metal halide bulb and a white light delivery optical fiber was used, with a cooled charge-coupled device camera (CCD, Hamamatsu C4742-95/C8484-03G02). White light was used in coordination with filters corresponding to the required acquisition channel. The devices were controlled by custom-made scripts in the interface of the micromanager software. The objectives were selected based on the required field of view and structure resolution. For ring closure experiments Olympus phase contrast air objectives 20X (N.A 0.4) or 40X (N.A 0.9) were used. The acquisition was done with 500 ms exposure and a 1 min frame interval to capture the temporal dynamics. To prevent evaporation of the media, the samples were covered with clean plastic lids, and the incubation chamber was maintained humid by constant storage of water inside the temperature-controlled microscope setup.

#### Laser ablation and fluorescence recovery after photo bleaching

Laser ablation experiments were performed using a TCS SP5 inverted microscope setup with a temperature-controlled chamber and a z-galvo stage. A pulsed multi photon laser (Coherent chameleon Ultra II; 800 nm; 4 MW) was used in synchrony with the TCS SP5 FRAP module. Pre- and post-ablation, the frame interval was set to 1.2 s. FRAP experiments were performed using a confocal spinning disk microscope (Nikon; Yokogawa CSU-X1 Confocal Scanner; Roper iLas FRAP system) for high-speed multi-dimensional acquisition. The objective was 100X Oil APO N.A 1.49. Bleaching was achieved using a visible light laser (561 nm) and the recovery was acquired using both 488 nm and 561 nm laser lines to image E-cadherin:mNG and MRLC::KO1 respectively. The acquisition was done using Photometrics Evolve 512 back-illuminated EMCCD.

#### Fluorescence resonance energy transfer (FRET)

Inverted Leica confocal microscope (SP5) equipped with a Z-galvo stage, MP excitation, and temperature control (whole microscope). A standard or resonant scanner (8000 Hz, 8000 lines/s) was used for fast imaging. Argon laser (458 nm, 40%) was used for excitation of the stoichiometric FRET couple (CFP/YFP) expressing cells. CX PL APO 40x/1.25–0.75 OIL objective was used for acquisition. The acquisition was done with photo-multiplier tube (PMT) detectors at an interval of 1 min. Quantitative ratiometric analysis (Donor/acceptor) of images was done using MATLAB software from Danuser Lab.^33^

#### Stimulated emission depletion microscopy (STED)

Post immunostaining, cells were imaged on an STED imaging setup equipped with confocal imaging. This was performed using an Abberior 3D-2 Color-STED system (Abberior Instruments, Göttingen) with a 100X N.A 1.4 oil objective (Olympus). Star580 was imaged with a pulsed laser at 560 nm, and excitation of Abberior Star Red and SiR-actin probe was performed at 640 nm. The depletion laser for both colors was a 775 nm pulsed laser (Katana HP, 3W, 1 ns pulse duration, NKT Photonics). STED images were acquired in Honigmann Lab. at MPI CBG Dresden.

#### Myosin cluster and ring analysis and statistics

Ring diameters and intensity profiles were measured with ImageJ. The onset of the analysis (t = 0) was taken from the start point where the diameter was 50 ± 2 μm. Speeds were computed through changes in diameters over time steps of 30 min. Clusters with a lifetime of shorter than 6 min were not used for analysis. The cluster contrast was determined by dividing the intensity of a cluster by the mean intensity of the whole ring perimeter. The cluster density depicts the number of clusters per ring perimeter averaged over time. A cluster was determined by using kymographs and tracing the clusters via Cartesian coordinates. Retraction velocity after laser ablation data was determined by calculating the distance between the retracting fronts over time. The computation of the velocity field of cell movement with respect to the cluster movement was done using the Particle Image Velocimetry (PIV) technique utilizing PIVlab open-source software. For correlative analysis of the FRET-MRLC signal, we sliced the ring along its perimeter in sections of 0.5 μm × 1 μm (radial x tangential). In each section, we measured the average MRLC density and the average FRET activity and normalized values for each ring section with respect to the ring average. To distinguish perimeter slices containing clusters from others, we manually tracked radial and tangential clusters using the Manual Tracking plugin in ImageJ.

### Quantification and statistical analysis

Statistical details can be found in Figure captions. The statistical significance was tested on OriginPro with a one-way analysis of variance and accepted at *p* < 0.05.

#### Analysis of laser ablation experiments

Acto-myosin ring and myosin clusters consist of a dynamic network of crosslinked actin and myosin filaments, generating active stresses within this network. Hence, post ablated retracting acto-myosin ring fronts behave as active viscoelastic material.^34^ Such viscoelastic material shows elastic behavior at short time scales. Therefore, the retraction velocity upon ablation corresponds to stress stored within the acto-myosin ring and is representative of total mechanical tension prior ablation. We measured the retraction velocity in radial clusters, in tangential clusters, and in the absence of clusters. We also measured the response of purely tangential clusters on wound rings with pillars left inside the monolayer. To evaluate tension corresponding to radial and tangential clusters, data were fitted with nonlinear exponential decay curve given by the equation: $=-\frac{T0}{\zeta }e^{-t/\tau }$ , with v the velocity of closure, T_0_ is the tension prior ablation and ζ is the damping coefficient.^16^ ζ characterises the frictional loss during interaction of the acto-myosin network with the surrounding fluid. We expect no changes in the value of ζ. Hence, ratio of initial velocities ν equates to ratio of tension and acts as a readout for the stress associated with distinct cluster types.

## Supplemental information

Document S1. Figures S1–S11

Video S3. Motion of clusters with respect to cell-cell junctions Cells express MRLC-KO1 (red) and E-cadherin mNG (green). Time in hh:mm:ss.
