# Supplementary material for: Myosin cluster dynamics determines epithelial wound ring constriction
Source: iScience. 2025 Jun 30;28(8):113030. doi: 10.1016/j.isci.2025.113030 (PMC12341579; doi:10.1016/j.isci.2025.113030)
Supplement: Document S1. Figures S1–S11 [file mmc1.pdf]

iScience, Volume *28*

Supplemental information

Myosin cluster dynamics determines epithelial wound ring constriction

Alka Bhat, Rémi Berthoz, Simon Lo Vecchio, Coralie Spiegelhalter, Shigenobu Yonemura, Olivier Pertz, and Daniel Riveline

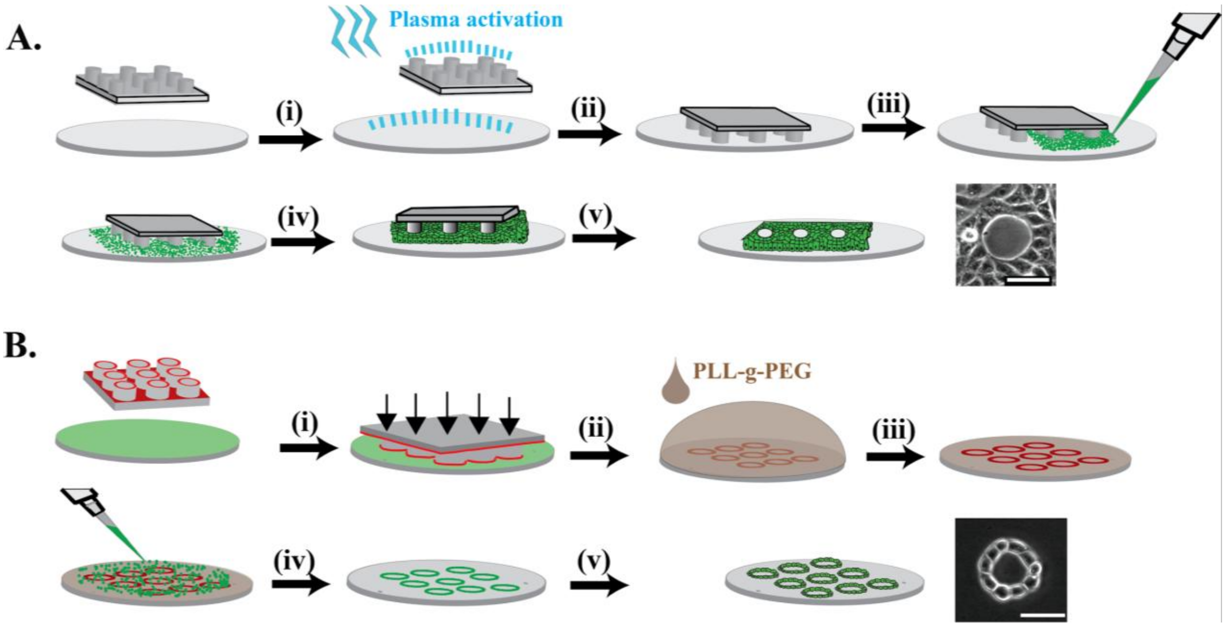

Figure S1. Schematic representation of protocols used to obtain multicellular rings : (A) Formation of rings in cell monolayer is obtained by (i) first the glass coverslip and the PDMS stamp with pillars is plasma activated, carefully. (ii) Next, they are bonded with each other and left at room temperature for 1h. (iii) After passivation with Pluronic acid for 1h, cells are introduced from all sides to ensure an even distribution, avoiding the pressure difference. (iv) Next, the assembly is incubated for 12-16h at 37°C and 5% CO_2 for cell spreading. (v) After removal of the PDMS pillars, reproducible shape controlled wound rings can be obtained (scale bar 50 µm). (B) Formation of single cell layer doughnut shape rings obtained by first incubating the PDMS stamp with fibronectin solution (100 µg/mL) for 30 min. This is followed by (i) bonding this PDMS stamp to Piranha cleaned and silanized coverslip. A weight is put on top of it, for an evenly distributed fibronectin stamping. This gives a glass coverslip stamped with fibronectin doughnut shape rings. (ii) This fibronectin stamped glass coverslip is next incubated with PLL-g-PEG (100 µg/mL) for 20 min to (iii) passivate the surface outside and inside the fibronectin doughnut shape rings. (iv) Next, epithelial (MDCK) cells are seeded on the coverslip and incubated for 1 h in low serum (1 % FBS) DMEM culture medium. After 1 h, the coverslip is washed with a new medium to remove the loosely attached cells settled on the passivated parts of the coverslip. (v) Next, the cells are allowed to spread for 30 min inside an incubator maintained at 37 °C with 5 % CO_2, forming epithelial doughnut shape rings (scale bar 50 µm).

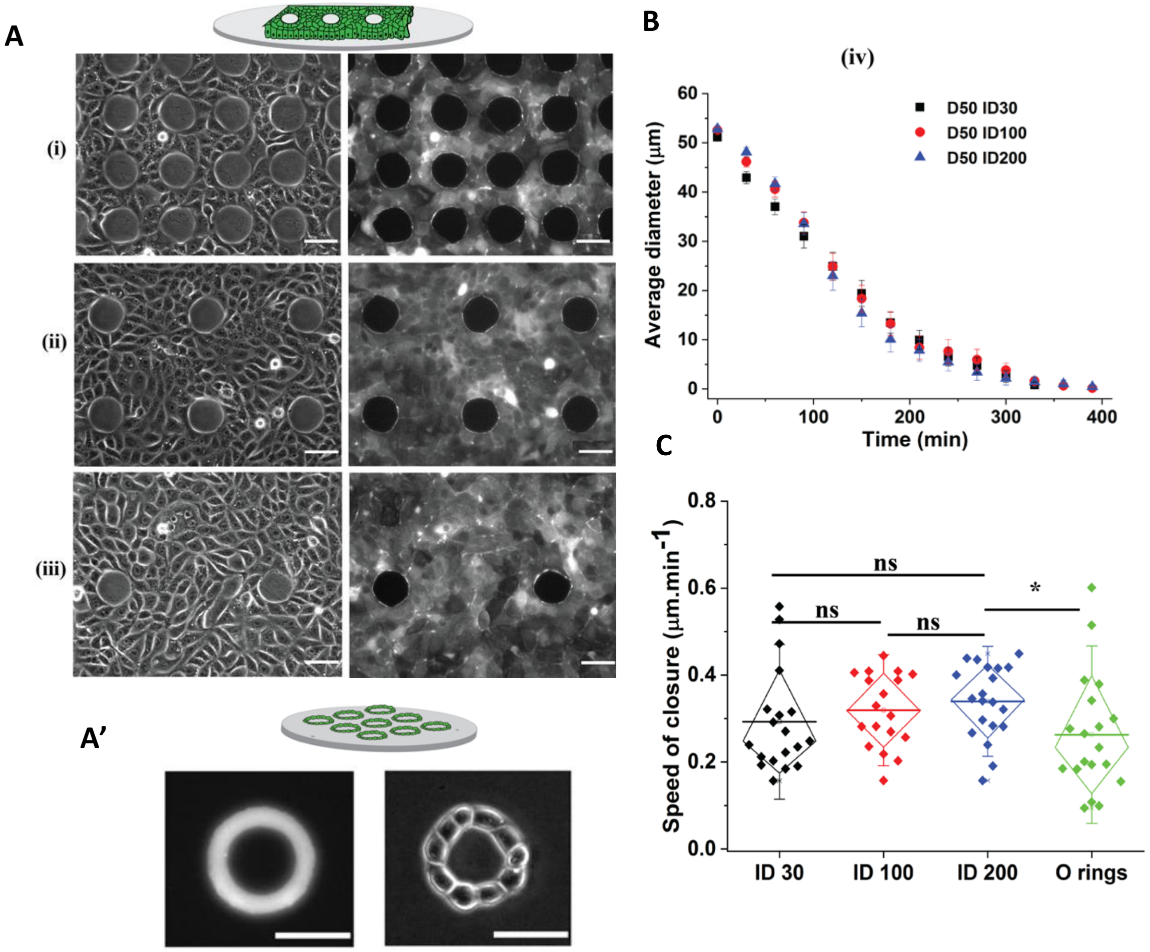

Figure S2: Velocity of closure in different conditions : closure is shown here as phase contrast (left panel) and fluorescent myosin (right panel, GFP): (A) We varied the distance between rings (i: 30 µm, ii: 100 µm and iii: 200 µm) keeping the cell density constant (scale bar: 50 µm). (A') Myosin fluorescence images of doughnut shape rings of cells (O rings) (scale bar: 50 µm). (B) Diameter of rings as a function of time for all conditions (error bars indicate SEM, n=3, 18 rings per condition). (C) We extract the speed in the linear part of each graph in (B). Each point represents speed of closure of a single ring in its corresponding condition. The mean closure speed (horizontal line) is comparable in all conditions: ~0.3 µm/min. Error bars: s.d., Test: 2-way ANOVA *p>0.05, n=3, 18 rings per condition.

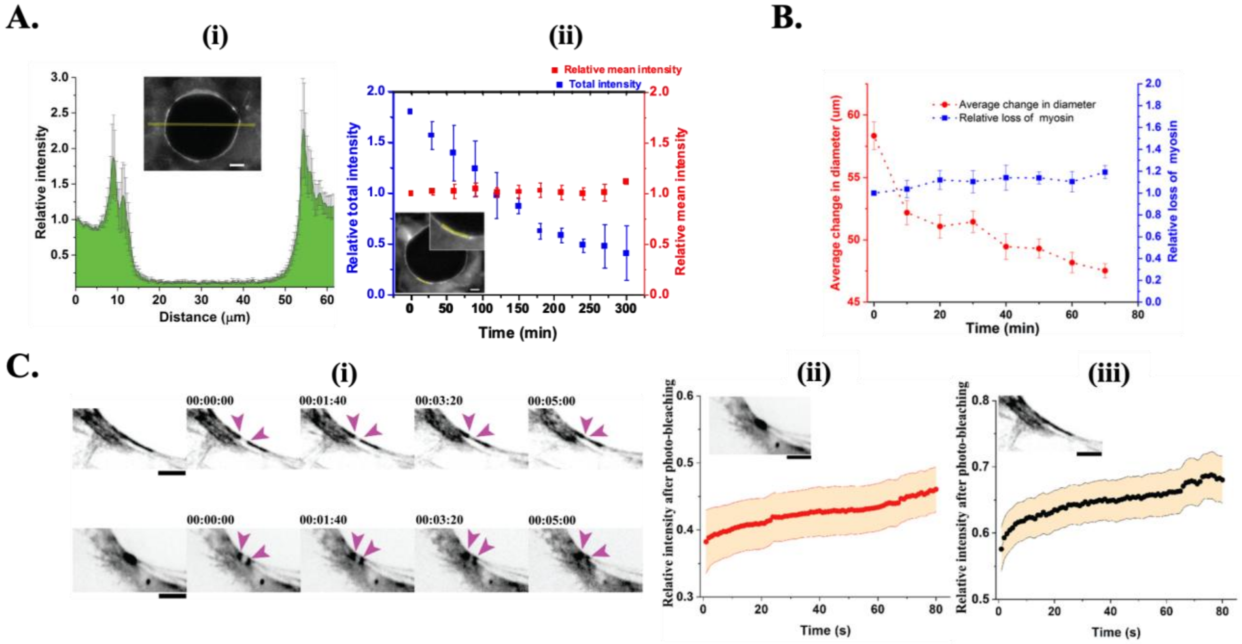

Figure S3. Myosin conservation and turnover in the acto-myosin ring: (A(i)) Myosin intensity profile across the wound, showing peaks in the acto-myosin cable (error bars: SEM, data from n=12 rings). Intensity data is normalized relative to the basal myosin levels at the ring periphery (extreme left and right pixels). The yellow line in the inset represents the line used to extract graph data. The insets show myosin fluorescence (scalebar: 10 μm). (A(ii)) Relative total (blue) and mean (red) intensities of the ring over time during constriction (error bars: SEM, data from n=3, 50 rings). The insets show myosin fluorescence (scale bar: 10 μm). (B) Myosin conservation evaluated by plotting relative myosin loss (blue) and change in diameter (red) during ring constriction, showing a constant myosin loss and therefore conservation of myosin (error bars: SEM, data from 4 rings). (C(i)) Myosin turnover evaluated by photobleaching the acto-myosin ring (top) as well as a myosin cluster (bottom), followed by fluorescence recovery between the bleached ends (magenta arrowheads) (inverted contrast, time code in hh:mm:ss, scale bar 5 μm). (C (ii-iii)) Fluorescence recovery evaluated over time for the cluster (ii) and the ring (iii) showing faster myosin turnover at the ring as compared to the cluster (data from n=3, 13 rings, 9 clusters). Insets show myosin fluorescence (inverted contrast, scale bar: 5 μm).

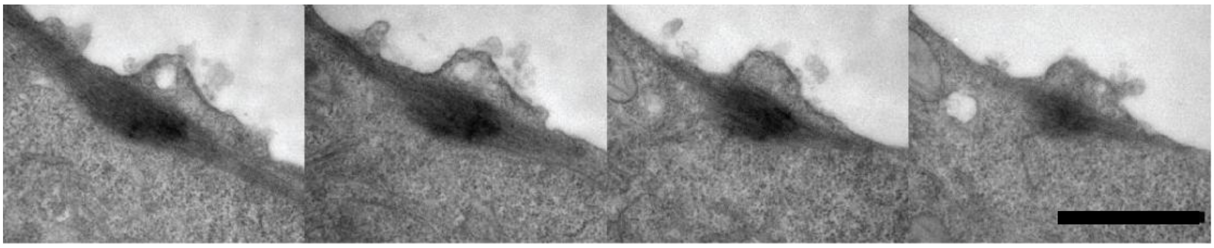

Figure S4. Electron microscopy of myosin clusters: Cluster sections with increasing distance of ~80 nm from the bottom of the coverslip depicted in the form of montage (left to right); see also Movie S8 and Movie S9. Scale bar: 1 µm.

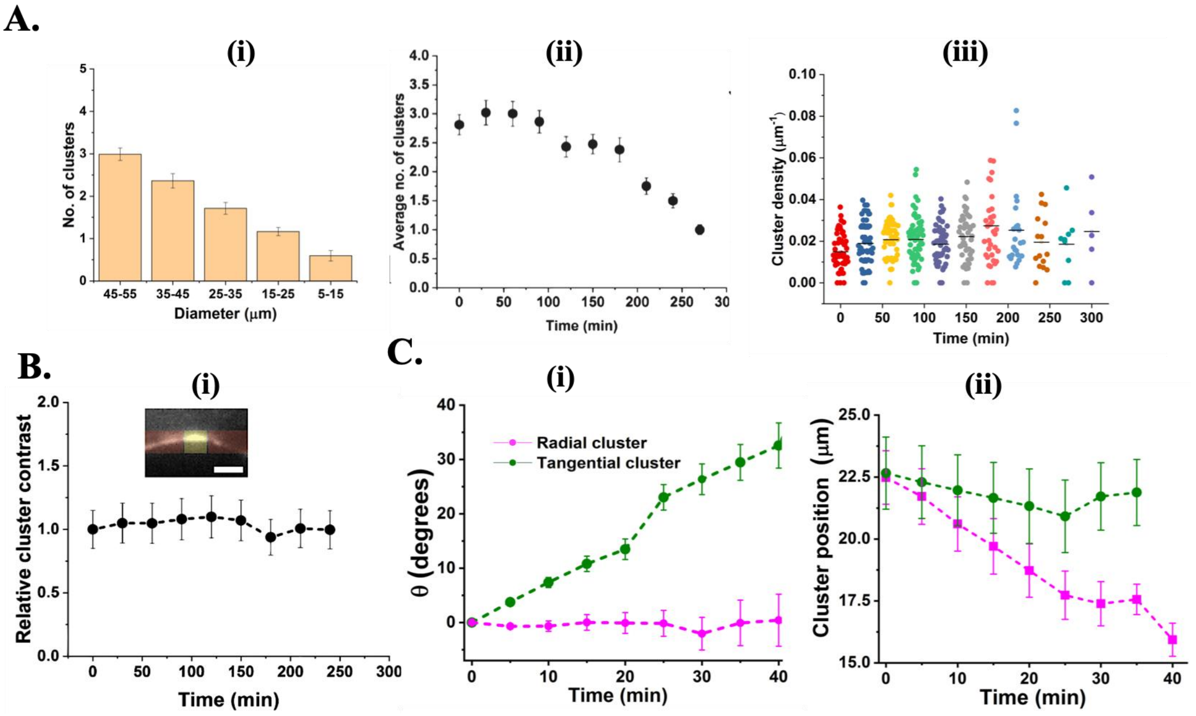

Figure S5. Myosin cluster density and characterization, at the wound ring perimeter: A. (i) Absolute cluster number and (ii) average cluster number, follows a decreasing trend corresponding to closing rings and/with increasing time, respectively. (iii) Cluster density evaluated to be constant across different time points Error bars SEM, n=3/N=55 rings. B. (i) Cluster (inset: yellow) contrast evaluated by intensity calculations of the cluster with respect to the ring background (inset: red) during constriction. The relative values were calculated with respect to the first time point. Error bars: SEM, n=3/ N=44 rings, scale bar 5μm. C (i): Angular displacement of clusters on a ring over time. Radial clusters (violet) do not change angular position compared to tangential clusters (green). During ring constriction. C (ii): Displacement of clusters on a ring over time. Radial clusters (violet) driving by wound closure move more than tangential clusters (green).

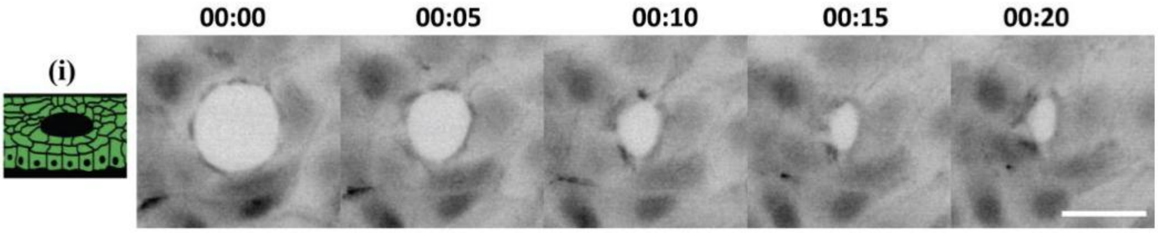

Figure S6. Myosin-II dynamics in wound rings expressing non-phosphorylatable MRLC (AA): Inverted look up table (LUT) of cells expressing MRLC AA-GFP showing reduction of myosin clusters during wound ring constriction. Scale bar 20 µm.

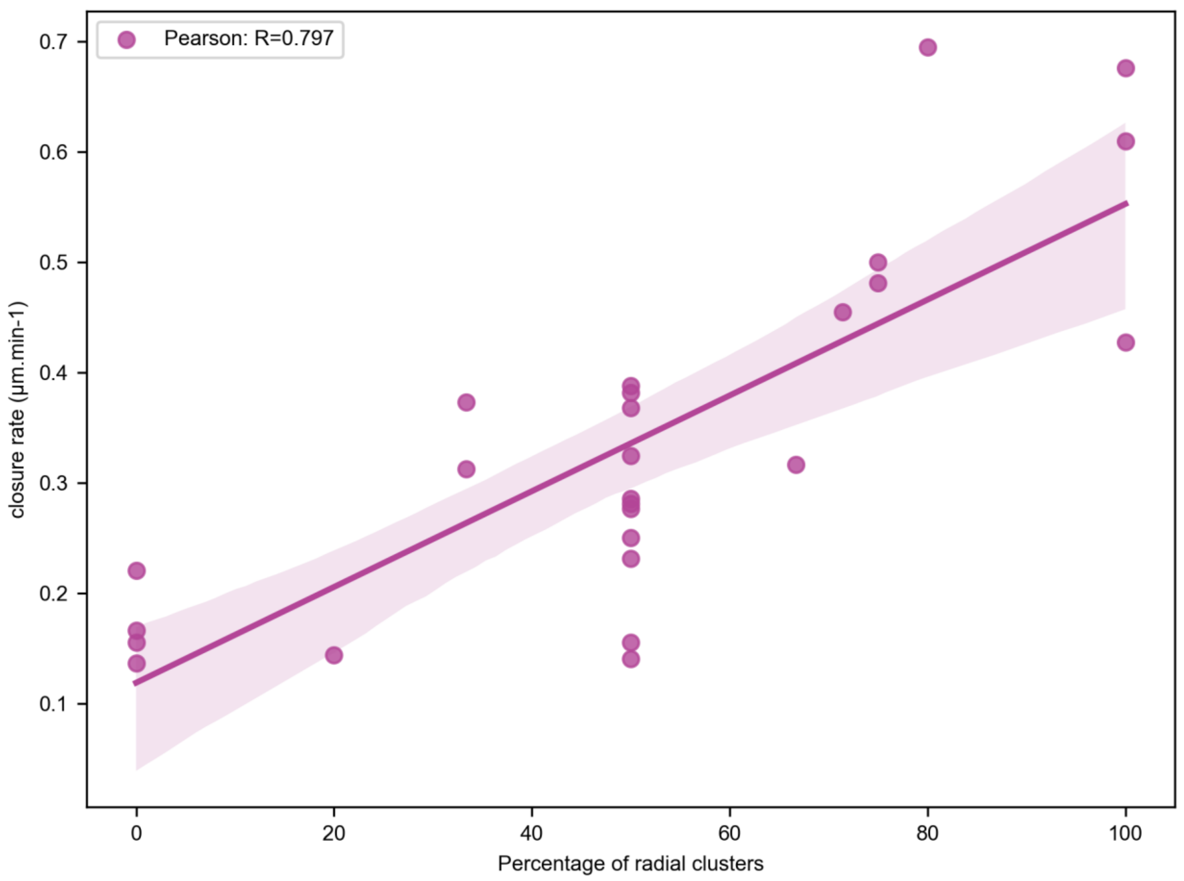

Figure S7. Correlation of wound closure velocity with percentage of radial clusters on the ring. We measured the number of radial clusters (out of the total number of clusters) on wound rings, and we measured the closure velocity. A high percentage of radial clusters correlates with fast wound closure (data from 26 wounds, 75 clusters).

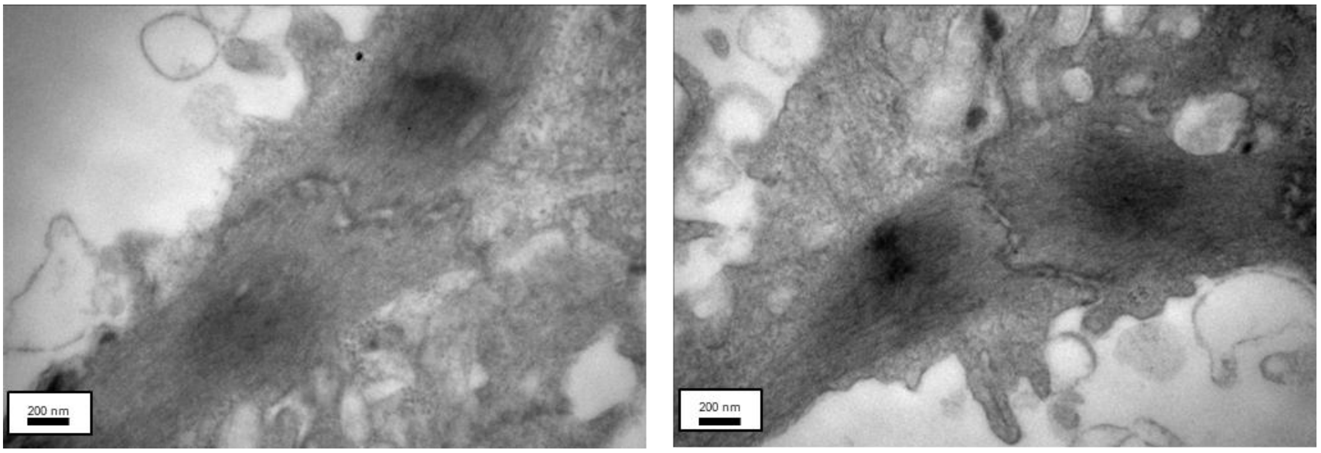

Figure S8. Cell-cell junction deformation in the vicinity of myosin clusters.

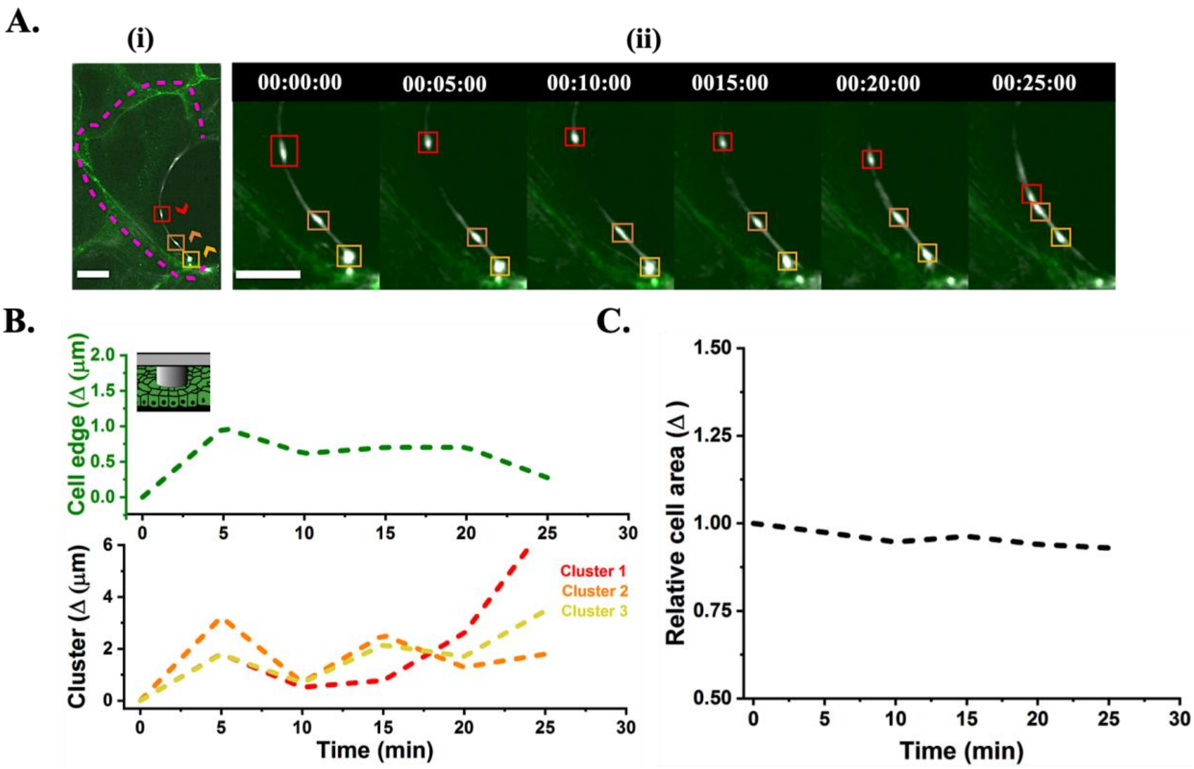

Figure S9. Dynamics of myosin clusters w.r.t the corresponding cell-cell junction: Movement of myosin clusters and the cell edge (i.e. cell-cell junction) was visualized by MRLC-KO1 (Kusabira orange) and Ecad-mNG (Neon green) respectively. A. (i) Initial state of three different clusters and their direction is indicated by color boxes and arrow heads respectively (red, orange, and yellow, one color per cluster). (ii) Corresponding dynamics of the clusters are tracked and depicted with their respective colored boxes. B. (Upper panel) Change in position of the cell edge was determined for the entire time period with respect to the reference (00:00:00). (Lower panel) Corresponding change in position of cluster 1 (red) 2 (orange) and 3 (yellow) was analysed with respect to to their initial positions showing dynamics independent of the cell edge movement. C. Cell area relative to the first time point (00:00:00) was shown to be constant with time. Time in hh:mm:ss. Scale bar:10 µm.

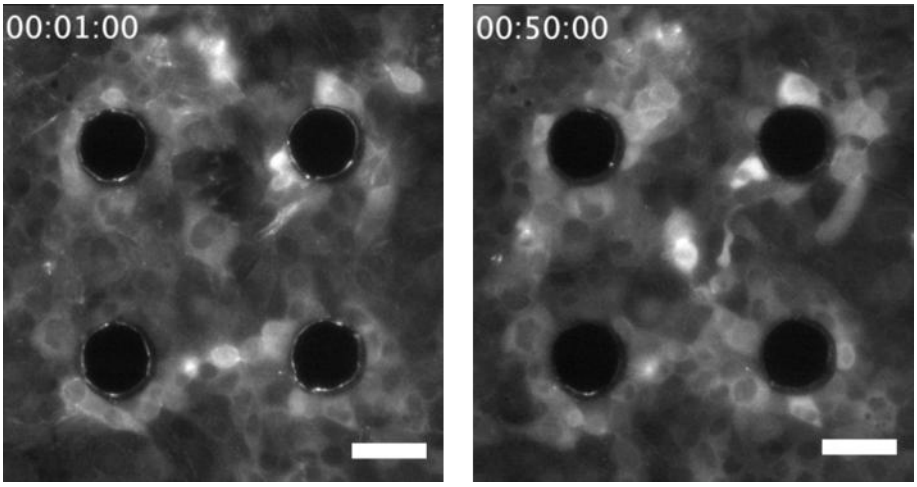

Figure S10. ROCK inhibitor Y27632 experiment. Clusters start disappearing after drug treatment.

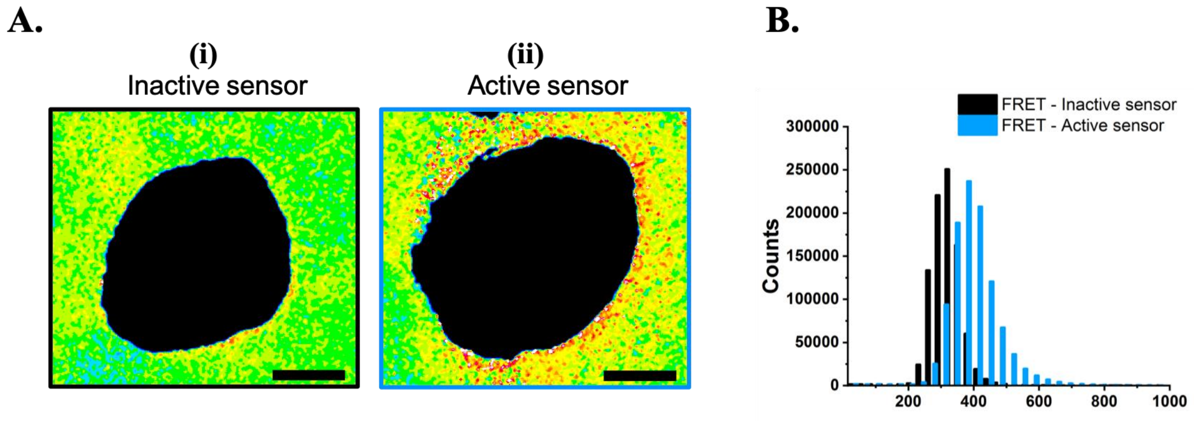

Figure S11. Comparison of intensity histograms between cells with active and inactive biosensor: A. Ratiometrically analysed images of monolayer area surrounding the wound rings in case of cells containing the (i) inactive biosensor and the (ii) active biosensor. B. Corresponding shift in histogram seen for the inactive biosensor (black) as compared to the active biosensor (cyan). Scale bar 10 µm. n=3/N=6 rings per condition
